# Delayed onset postoperative retropharyngeal hematoma after anterior cervical surgery with a sequela of tracheal stricture: a case report

**DOI:** 10.1186/s40001-021-00550-6

**Published:** 2021-07-20

**Authors:** Dong-Gune Chang, Jong-Beom Park, Hong Jin Kim, Soo-Bin Park

**Affiliations:** 1 Department of Orthopaedic Surgery, Inje University Sanggye Paik Hospital, College of Medicine, Inje University, Seoul, Korea; 2 Department of Orthopaedic Surgery, College of Medicine, The Catholic University of Korea, Seoul, Korea; 3 Department of Orthopaedic Surgery, Uijeongbu St. Mary’s Hospital, The Catholic University of Korea, 271 Cheonbo-ro, Uijeongbu-si, 11765 Gyeonggi-do Korea

**Keywords:** Delayed onset, Postoperative retropharyngeal hematoma, Tracheal stricture, Anterior cervical surgery

## Abstract

**Background:**

Among the several complications associated with anterior cervical discectomy and fusion (ACDF), airway compromise is considered one of the serious life-threatening conditions and usually requires emergent treatment, including airway establishment and hematoma evacuation surgery. Postoperative retropharyngeal hematoma commonly occurred during the on immediate phase with airway compromise, but have a rarity on late onset of this complication. Enlightened by this existing fact, we report the first case of delayed onset postoperative retropharyngeal hematoma after anterior cervical surgery with a sequela of tracheal stricture.

**Case presentation:**

A 55-year-old male underwent ACDF for disc herniation at C5–6 that had not responded to conservative treatment over 3 months. The symptoms significantly improved after surgery, and he was discharged on postoperative day 3. On the 7 days after ACDF, the patient complained of sudden-onset left-deviated neck swelling. The follow-up plain radiographs and neck-enhanced computed tomography (CT) scans showed anterior and right lateral displacement of the airway including the trachea by a large retropharyngeal hematoma. We performed an emergent forceful endotracheal intubation that was maintained for 2 days until the patient underwent hematoma evacuation surgery. On the second day after hematoma evacuation surgery, the patient complained of hoarseness with a foul breath odor. Laryngoscopy showed tracheal ischemic mucosal damage that had been induced by forceful endotracheal intubation. Antibiotics and systemic corticosteroids were administered, and the symptoms improved. One month after hematoma evacuation surgery, he complained of dyspnea on exertion, and laryngoscopy showed tracheal stricture. The patient underwent bronchoscopic dilatation and is doing well without recurrence of symptoms.

**Conclusions:**

Early surgery to remove the delayed onset retropharyngeal hematoma, rather than forceful endotracheal intubation followed by delayed surgery, might yield better results and avoid unexpected complications of tracheal stricture.

## Background

Anterior cervical discectomy and fusion (ACDF), one of the most common anterior cervical surgeries, has been widely used for patients with degenerative cervical diseases or trauma, and it achieved satisfactory clinical and radiological outcomes [1]. Several postoperative complications after ACDF, however, can occur. A high index of suspicion and special attention are needed to avoid life-threatening complications, such as airway compromise due to acute onset of postoperative retropharyngeal hematoma [1, 2]. According to previous studies, acute-onset postoperative retropharyngeal hematoma usually occurs 6 to 72 h after surgery [3, 4]. The frequency of late-onset postoperative retropharyngeal hematoma is very rare, and this complication has been mainly described in the form of case reports. Nonetheless, no studies have previously reported delayed onset postoperative retropharyngeal hematoma associated with a sequela of tracheal stricture requiring bronchoscopic dilatation. Therefore, we present and discuss the first case of tracheal structure as a sequela of delayed onset postoperative retropharyngeal hematoma that occurred in a patient more than 7 days after ACDF.

## Case presentation

**Fig. 1:**
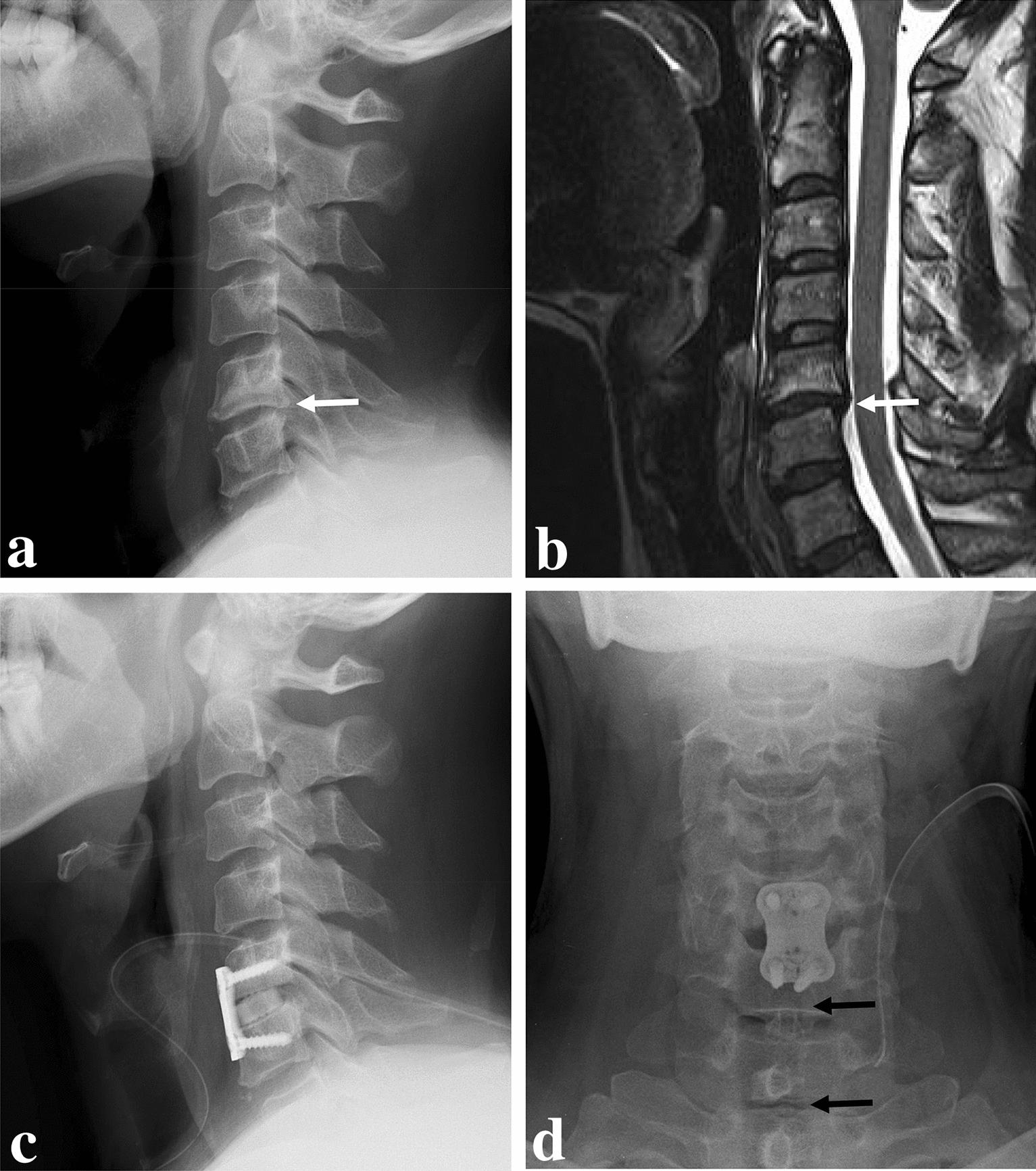
Lateral radiograph (**a**) and sagittal magnetic resonance imaging (**b**) showed disc space narrowing, posterior osteophyte and disc herniation (white arrows). Postoperative lateral (**c**) and anteroposterior (**d**) radiographs showing anterior cervical discectomy and fusion with plating and a normal tracheal shadow (dark arrows)

A 55-year-old male complained of posterior neck pain with pain and tingling sensation in the left arm that had not responded to conservative treatment over the previous 3 months. He had no definite history associated with a bleeding tendency. The neurological examination was within the normal limit without pathologic reflex. Preoperative radiographs and sagittal magnetic resonance imaging (MRI) showed disc space narrowing, posterior osteophyte, and disc herniation at C5–6 (Fig. 1a and b). The patient underwent ACDF with plating at C5–6, and a normal tracheal shadow was observed on immediate postoperative radiographs (Fig. 1c and d). The patient’s radiculopathy symptoms improved significantly after surgery, and he was discharged on postoperative day 3.

**Fig. 2:**
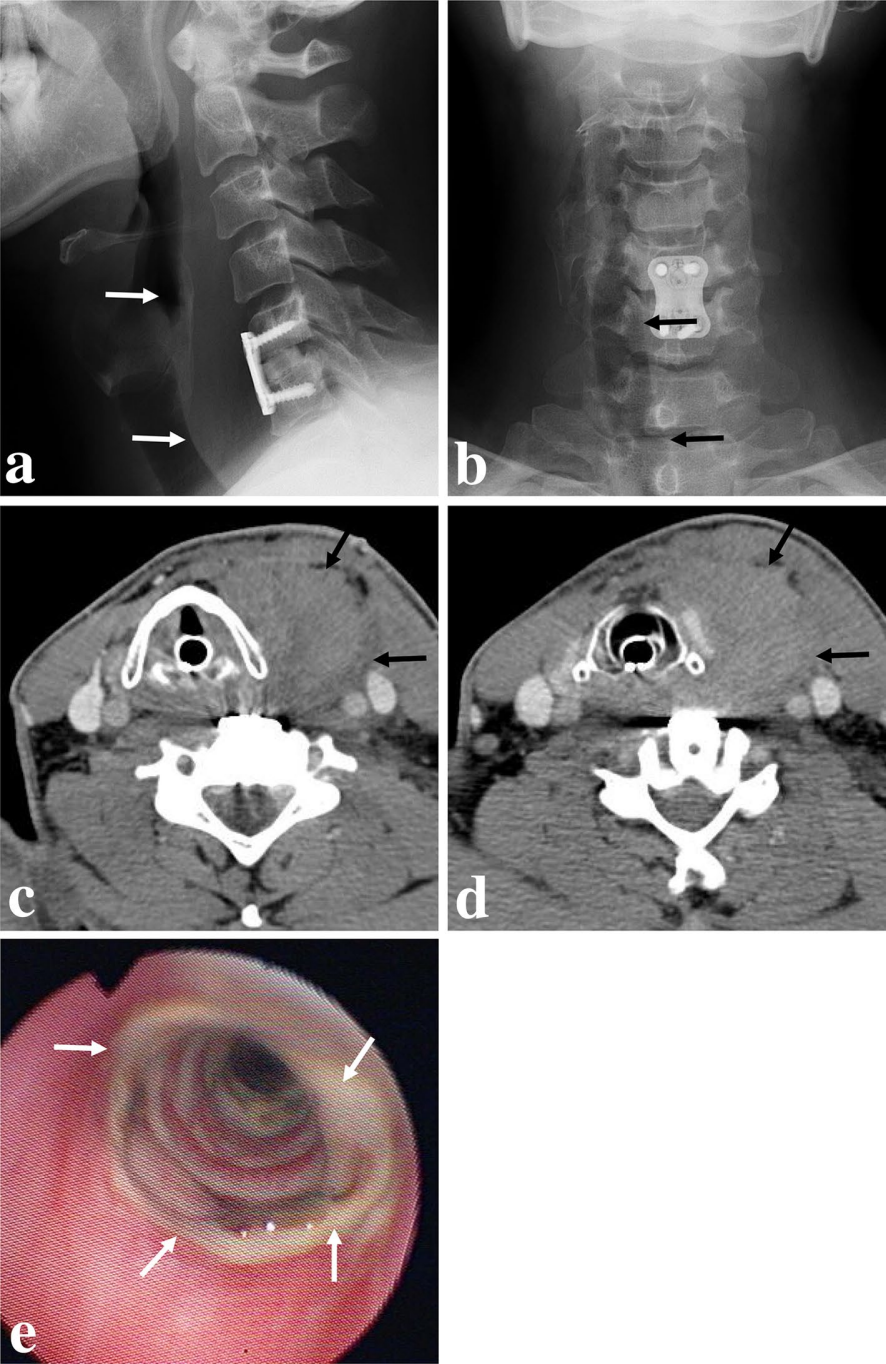
Seven days after surgery, follow-up lateral (**a**) and anteroposterior (**b**) radiographs showed increased anterior soft tissue swelling (white arrows) and tracheal deviation to right side (dark arrows). Neck-enhanced computed tomography scans (**c** and **d**) showing anterior and right lateral displacement of the airway including the trachea by a large retropharyngeal hematoma (dark arrows). Laryngoscopy (**e**) showed tracheal granulation tissues (white arrows)

However, the patient visited the outpatient clinic 4 days after discharge (postoperative day 7) complaining of sudden-onset left-deviated neck swelling. The patient did not show any sign of respiratory difficulty (i.e., low O2 saturation and severe dyspnea). Follow-up radiographs showed increased anterior soft tissue swelling as well as a right-side-deviated trachea (Fig. 2a and b). The neck-enhanced computed tomography (CT) scans showed anterior and right lateral displacement of the airway including the trachea that was compressed by a large retropharyngeal hematoma (Fig. 2c and d). To prevent airway obstruction, we performed an emergent endotracheal intubation, and intubation was maintained for 2 days until the patient underwent hematoma evacuation with ligation of a small vessel located along the carotid sheath for control of the bleeding focus. The hemovac drain showed 10 cc at postoperative day 1, and 3 cc at postoperative day 2 with bloody aspect and no sign of infection. Two days after hematoma evacuation surgery, the hemovac drain was removed because the aspect of drain was bloody without infection signs. However, the patient complained of hoarseness with a foul breath odor after the removal of hemovac drain. Laryngoscopy showed granulation tissues in the trachea, likely due to tracheal ischemic mucosal damage caused by a forceful endotracheal intubation in which the trachea was severely deviated and compressed from large retropharyngeal hematoma (Fig. 2e). The patient received antibiotics (amoxicillin/clavulanate 3.6 g bis in die [B.I.D]) intravenously and systemic corticosteroids (prednisolone 30 mg B.I.D) orally for 1 week according to the instructions from an otolaryngologist. The patient’s symptoms improved, and he was discharged after 1 week.

**Fig. 3:**
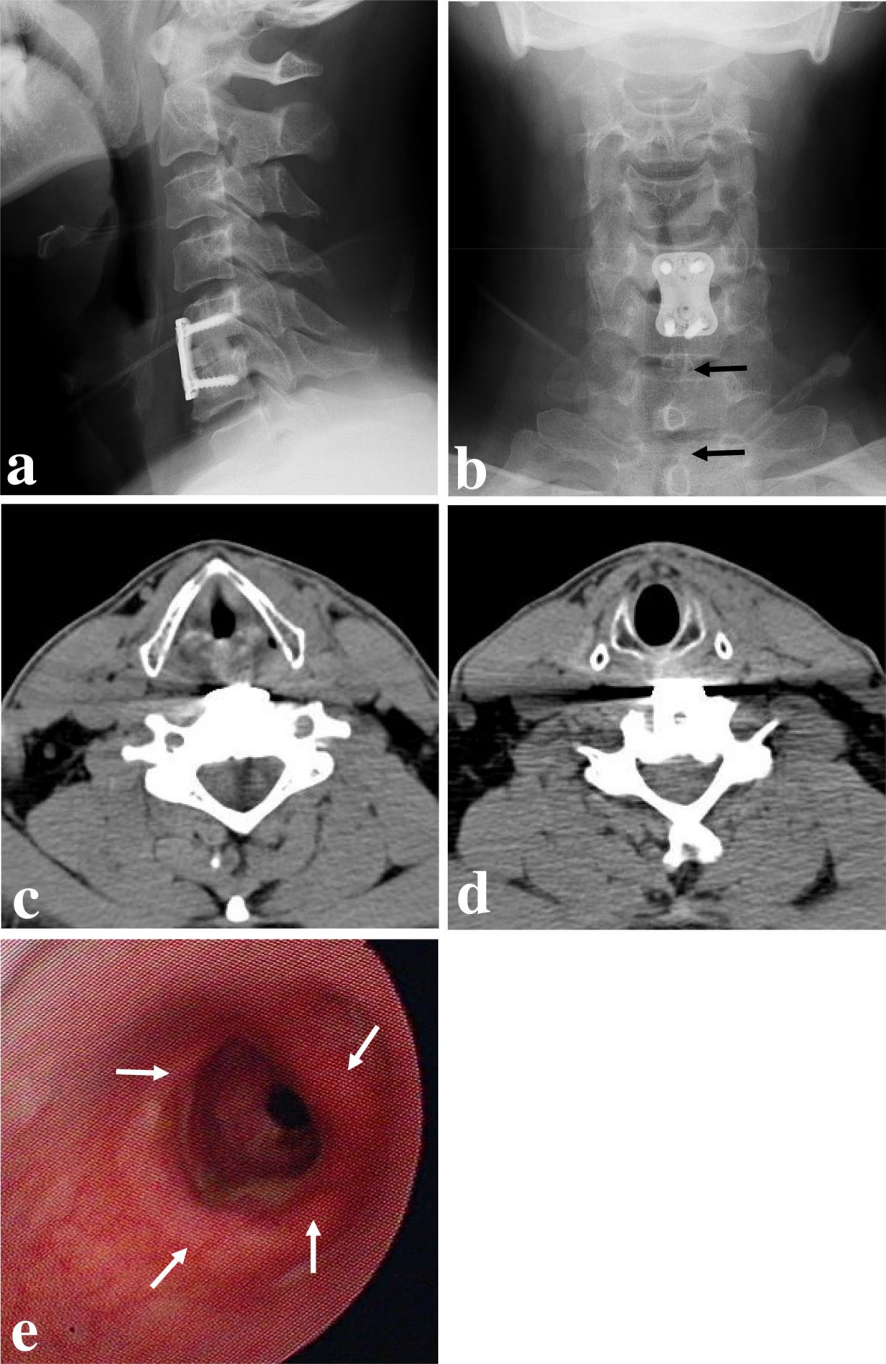
One month after hematoma evacuation surgery, follow-up lateral (**a**) and anteroposterior (**b**) radiographs showed normalization of the previous anterior soft tissue swelling and tracheal deviation (dark arrows). Follow-up neck-enhanced computed tomography scans (**c** and **d**) showing normalization of the previous anterior soft tissue swelling and tracheal deviation. Follow-up laryngoscopy (**e**) showed tracheal stenosis and stricture (white arrows)

One month after hematoma evacuation surgery, the patient revisited the outpatient clinic complaining of dyspnea on exertion. However, follow-up radiographs and neck-enhanced CT scans showed no definite recurrence of retropharyngeal as well as normalization of previous anterior soft tissue swelling and tracheal deviation (Fig. 3a–d). Laryngoscopy, performed to further evaluate the patient’s dyspnea, showed tracheal stenosis and stricture (Fig. 3e). The patient’s dyspnea symptoms did not improve after 2 months of conservative treatment. Therefore, the patient underwent bronchoscopic dilatation without stent insertion, and he is doing well without recurrence of re-stenotic symptoms from 2-year follow-up in outpatient clinic.

## Discussion and conclusions

ACDF is one of the most commonly performed surgical procedures for managing degenerative cervical diseases or trauma [1, 2]. Approximately more than 90% of patients reported being satisfied with the clinical and radiological results following ACDF. Therefore, it has been widely accepted as the gold standard treatment of cervical radiculopathy, myelopathy, and trauma [2]. However, according to multiple previous studies, dysphagia (1.7 to 9.5%), postoperative retropharyngeal hematoma (1.3% to 5.6%), respiratory insufficiency (1.1%), and esophageal perforation (0.3 to 0.9%) contribute to the morbidity rates for ACDF, which vary from 13.2% to 19.3% [5]. Among the several complications associated with ACDF, airway compromise is considered one of the serious life-threatening conditions and usually requires emergent treatment, including airway establishment and hematoma evacuation surgery [6, 7]. The risk factors for a second hematoma evacuation surgery include three or more level surgeries, obesity, anemia, use of anticoagulation agents, and male gender [5]. In our case, there were no related risk factors except male gender.

Lied et al. found that postoperative retropharyngeal hematoma commonly occurred during the on immediate phase (0 to 6 h) with airway compromise [8]. O’Neill et al. reported that 65% of postoperative retropharyngeal hematomas occur within 24 h, and 35% within 6 days [9]. Therefore, late onset of this complication is rare, as shown in our unique case. Regarding postoperative airway compromise following ACDF, predictable etiologies by time were reported as follows: angioedema (6 to 12 h), retropharyngeal hematoma (12 to 24 h), pharyngolaryngeal edema (24 to 72 h), and retropharyngeal abscess (72 to 96 h) [4]. Generally, emergent evacuation should be recommended for retropharyngeal hematoma at the time of diagnosis because it can cause life-threatening events due to acute airway compromise. In our case, retropharyngeal hematoma and edematous changes mechanically compressed the trachea, resulting in anatomical deviation and distortion. Furthermore, an attempt to secure the airway by forceful endotracheal intubation caused severe trachea mucosal damage, and the unforeseen consequences included tracheal stenosis, which required bronchoscopic dilatation. Therefore, timely and appropriate urgent intervention at the diagnosis of retropharyngeal hematoma is very important to prevent further unforeseen complications.

Tracheal stricture and stenosis are sequalae of complications from endotracheal or translaryngeal intubation and cause serious problems, such as dyspnea on exertion [10]. The incidence of tracheal stricture and stenosis varies according to the duration of endotracheal intubation and severity of trachea mucosal damage [11]. In general, the severity of tracheal stricture and stenosis depends on the severity of trauma at the time of endotracheal intubation, high balloon pressure, and excessive movement during the intubation period [12]. Only 2% of cases with endotracheal intubation less than 6 days reported tracheal stricture and stenosis. In our case, although the duration of endotracheal intubation was just 2 days, tracheal stricture and stenosis occurred secondary to tracheal mucosal damage. We hypothesize that forceful endotracheal intubation caused excessive tracheal mucosal damage, resulting in tracheal stricture and stenosis, in a patient with a mechanically compressed and deviated trachea caused by a large retropharyngeal hematoma [13]. The most common site of tracheal mucosal damage is the endotracheal tube cuff site because pressure from the cuff on the tracheal wall can cause loss of local blood flow [12, 13]. Bronchoscopy is the mainstay of diagnosis for tracheal mucosal damage and stricture. In addition, bronchoscopic dilatation and/or stent insertion can be useful [12]. In the benign condition, benefit of endotracheal stent showed less clear because of long-term prognosis, difficulty of removing stents, and complications. In our case, considering recurrent infection and granulation tissue formation from the insertion of endotracheal stent, the otolaryngologist firstly considered brochoscopic dilatation only to resolve dyspnea caused by tracheal stenosis [14].

In cases of delayed onset retropharyngeal hematoma, our case suggests that emergent hematoma evacuation is a better treatment choice than delayed surgery with forceful endotracheal intubation to avoid the complications of tracheal stricture and stenosis. However, a practical reality is that emergent hematoma operation is not always feasible from a logistical standpoint. Intubation in our case was forcefully performed to prevent airway obstruction because of several practical problems at the hospital such as securing an operating room, time to wait for MRI and CT examination, and noting per oral (NPO) status of patient. Therefore, if emergent surgery was practically possible, hematoma evacuation should be performed as quickly as possible after diagnosis.

Currently, the guidelines for the management of acute-onset postoperative retropharyngeal hematoma are not well established [15]. Several cases of retropharyngeal hematoma have been successfully resolved by providing oxygen with the patient in the sitting position [16]. However, only patients with stable vital signs and no definite severe respiratory compromise symptoms, such as stridor, swelling, and cyanosis, can be considered for conservative management [2]. Many studies recommend emergent hematoma evacuation surgery for rapid improvement of symptom and prevention of potential life-threatening events. However, no guidelines for the management of delayed onset retropharyngeal hematoma have been established. Therefore, our case is thought to be helpful in determining the appropriate treatment for delayed onset retropharyngeal hematoma.

In conclusion, our case of delayed onset retropharyngeal hematoma showed that forceful endotracheal intubation can cause tracheal ischemic mucosal damage, which can result in tracheal stricture requiring bronchoscopic dilatation. In cases of delayed onset retropharyngeal hematoma, early surgery to remove the hematoma might yield better results and avoid unforeseen complications of tracheal stenosis and stricture.

## Data Availability

Not applicable as this is a case report.

## Abbreviations

ACDF: Anterior cervical discectomy and fusion
CT: Computed tomography
MRI: Magnetic resonance imaging
